# Correlation between Adenomyosis and Endometrial cancer: 6-year experience of a single center

**Published:** 2018-09

**Authors:** OD Zouzoulas, D Tsolakidis, I Efstratiou, S Pervana, E Pazarli, G Grimbizis

**Affiliations:** Department of Obstetrics & Gynecology, Aristotle University of Thessaloniki, “Papageorgiou” Hospital, Thessaloniki, 56403, Greece; Anatomical Pathology Laboratory, “Papageorgiou” Hospital, Thessaloniki, 56403, Greece

**Keywords:** Adenomyosis, concurrence, endometrial cancer, malignant transformation

## Abstract

**Introduction:**

Adenomyosis often co-exists in the pathological specimens after surgery for endometrial cancer. The aim of this study is to describe the clinicopathological and oncological characteristics of these patients and further investigate the possibility of malignant transformation in the adenomyotic tissue.

**Methods:**

We retrospectively reviewed the medical records of all patients that underwent hysterectomy for endometrial cancer (January 2012 – December 2017). The pathological reports were studied and when adenomyosis was present, the pathological slides were reviewed in order to discover any malignant change in the adenomyotic tissue. The clinicopathological characteristics and oncological results were described.

**Results:**

Out of 229 cases of endometrial cancer, 64 (28%) patients had concurrently endometrial cancer and adenomyosis. Among these 64 patients, 7 (11%) had malignant transformation of adenomyosis. The mean age of patients suffering from both endometrial cancer and adenomyosis was 63.2 years old and 57 (89%) of these patients, had early endometrial cancer. Concerning the patients with malignant transformation of adenomyosis, their mean age was 65 years old with no premenopausal case.

**Discussion:**

Adenomyosis has been described in the last decades, but its malignant transformation into endometrial cancer is not fully undercovered. Further investigation is needed in order to clarify the pathologic progression of adenomyotic lesions to endometrial cancer.

## Introduction

Endometrial cancer is the 6 th most common cancer in women worldwide and the 14 th most common cancer worldwide, with higher incidence in Northern America and Europe (Ferlay et al., 2015). Furthermore, it is the most common cancer among gynecological malignancies, with an increase rate of 1% - 2% annually (de la Orden et al., 2008; Zullo et al., 2005). The main prognostic factors of the disease are age, histological type, tumor grade, depth of myometrium invasion and lymph node involvement (Creasman et al., 2006). Recently, adenomyosis, is considered by some investigators as a precursor for endometrial cancer (Habiba et al., 2018).

Adenomyosis, as mentioned above, is a benign disease that is often diagnosed in the last decades due to the progress of the imaging techniques. It is characterized by the ectopic presence of endometrial tissue inside the myometrium, meaning that endometrial glands and stroma are surrounded by reactive smooth muscle cells (Bergeron et al., 2006). This condition is mostly found in reproductive age women (Bergeron et al., 2006) and the spectrum or severity of the clinical symptoms can vary from no symptoms (33%) to chronic pelvic pain (77%) and heavy menstrual bleeding (40 – 60%) (Struble et al., 2016). Commonly it co-exists in pathological reports of hysterectomy specimens with endometrial cancer, particularly endometrial histotype (Gizzo et al., 2016).

Sometimes the adenomyotic tissue might conceal premalignant or malignant transformation. One question that arises is if endometrial cancer, which arises within the adenomyotic tissue worsens the prognosis and survival of these patients. The aim of this study is to describe the clinicopathological and oncological characteristics of patients suffering simultaneously from these two pathologic entities, endometrial cancer – and adenomyosis, and further investigate the malignant transformation of the adenomyotic tissue.

## Methods

### Design, patients and inclusion criteria

We retrospectively reviewed the medical records of all patients (N=273) that underwent surgery for endometrial cancer from January 2012 until December 2017 in our clinic. After the inclusion and exclusion criteria for this study were set, we identified 229 patients eligible for further analysis. Inclusion criteria were:

- Preoperative histological confirmation of endometrial adenocarcinoma (curettage)
- Preoperative transvaginal ultrasound and detailed medical history
- Surgical treatment should include at least hysterectomy

Exclusion criteria were:

- Preoperative administration of chemotherapy or endocrine therapy
- Primary tumor in other organs of the body (e.g. ovarian cancer)
- Missing important data

The pathological reports of the selected patients (n=229) were extensively studied for adenomyosis, uterine fibroids and endometriosis. When adenomyosis was present, the relevant pathological slides were independently reviewed by two expert pathologists in order to discover any premalignant or malignant transformation in the adenomyotic tissue. Adenomyosis was defined as the presence of endometrial glands and stroma inside the myometrium, with a minimum distance of 4 μm from the endomyometrial junction (Vercellini et al., 2006). However, it is important to distinguish the cases where endometrial cancer invaded the myometrium and the adenomyotic tissue from cases where endometrial cancer co-exists with malignant transformation of the adenomyotic tissue.

All patients underwent hysterectomy with or without pelvic / paraaortic lymphadenectomy and cytological examination of pelvic washing. After the multidisciplinary team meeting, if it was necessary, patients also received adjuvant radiotherapy with or without chemotherapy and of course a close follow-up every 6 months. The clinicopathological characteristics and oncological results were evaluated, in order to understand clinical presentations and prognosis of the two concurrent diseases. Data selections included age, menopausal status, tumor grade and the stage of endometrial cancer, preoperative cancer antigen CA-125 levels and the presence or not of adenomyosis, uterine fibroids and endometriosis.

The patients were divided into two groups according to the coexistence or not of adenomyotic tissue. Group A included women suffering from both endometrial cancer and adenomyosis and Group B women, suffering from endometrial cancer alone. All the aforementioned data were statistically analyzed between the two groups and then Group A was further divided into two subgroups on the basis of malignant transformation of the adenomyotic tissue.

### Statistics

The statistical analysis was performed using RStudio. Continuous variables were checked for normality and the accordingly parametric or not parametric tests were applied. Moreover, categorical variables were presented as counts and percentages and chi-square of Fisher exact tests were used for the comparison between the two groups. All results were rounded to one decimal. Statistical significance was set at p-value < 0.05.

## Results

After reviewing the medical records of all patients included in this study, the main recorded symptoms of these women were either menstrual disorders, postmenopausal vaginal bleeding or chronic pelvic pain and according to the preoperative ultrasound, thickening of the endometrium was always noted. All 229 patients with endometrial cancer were divided into two groups based on the co-existence or not of adenomyosis: 64 (28%) patients had concurrently endometrial cancer and adenomyosis (Group A) and 165 (72%) patients had only endometrial cancer (Group B). The mean age of women included in Group B was 64.2 ± 12.3 years old and most of them, 138 (83.6%) were postmenopausal. One third of them, 56 (33.95), had concurrent uterine fibroids and suspiciously only 2 (1.2%) presented endometriotic lesions in the genital system. Most of them, 106 (77.4%), had CA125 levels (<35.0 U/ml) within the normal limits.

On the other hand, women from Group A (concurrent endometrial cancer and adenomyosis) had a mean age of 63.2 ± 9.4 years old and the majority of them, 56 (87.5%) were also postmenopausal. Uterine fibroids were present at 25 (39.1%) patients and again only 2 (3.1%) had endometriosis. CA125 levels were found increased (>35.0 U/ml) only in a few patients, 10 (19.6%), as well. The most important data, which play a prognostic role, were the stage of the disease at the time of the diagnosis and the tumor grade, which were retrieved. In Group A most of the patients, 57 (89%), had early endometrial cancer (FIGO stage IA-IB). This presented a statistically significant difference from Group B (p-value < 0.05), who presented with advanced endometrial cancer (FIGO stage II-III). Furthermore, from Group A, 22 (34.4%) women had grade I, 36 (56.2%) grade II and only 6 (9.4%) had grade III tumors. So, 2/3 of the patients presented with grade I-II tumors, which was statistically significant different compared to Group B patients (p-value < 0.05), who had more grade III tumors (40; 24.3%). All the above-mentioned data are presented in Table I.

**Table I:** Characteristics of patients with endometrial cancer

		EC with adenomyosis (N=64)	EC without adenomyosis (N=165)	P-value
Age (years)		63.2 ± 9.4	64.2 ± 12.3	0.26
Menopause		56 (87.5%)	138 (83.6%)	0.46
Co-existing Fibroids		25 (39.1%)	56 (33.9%)	0.46
Co-existing Endometriosis		2 (3.1%)	2 (1.2%)	0.31
CA125 elevated (>35 U/ml)		10 (19.6%)	31 (22.6%)	0.65
FIGO Stage				< 0.05
	I	57 (89%)	101 (61.2%)	
	II	3 (4.7%)	30 (18.2%)	
	III	3 (4.7%)	25 (15.1%)	
	IV	1 (1.6%)	9 (5.5%)	
Tumor Grade				< 0.05
	1	22 (34.4%)	38 (23%)	
	2	36 (56.2%)	87 (52.7%)	
	3	6 (9.4%)	40 (24.3%)	

Moreover, patients from Group A were divided into two groups for further analysis. GroupA1: 57 (24.9%) patients with endometrial cancer and adenomyosis. Group A2: 7 (3.1%) patients with concurrent endometrial cancer and malignant transformation of the adenomyotic tissue. The slides were independently reviewed by two pathologists, in order to confirm that the cancerous originated from the malignant transformation of the adenomyotic tissue and not from the endometrial cancer invasion to the myometrium. The mean age of the patients with malignant transformation of the adenomyosis was 65 ± 10.1 years old with no premenopausal case. The majority, 6 (85.7%), of the patients had early endometrial cancer (FIGO stage I) with various tumor grades [grade I: 2 (28.6%), grade II: 3 (42.8%), grade III: 2 (28.6%)]. Only 2 (33.3%) cases had elevated CA125, not over 100 U/ml. There was no statistically significant difference between the two subgroups in any of the above-mentioned data. All results are shown in Table II.

**Table II:** Characteristics of patients with adenomyosis malignant transformation

		Adenomyosis without malignant transformation (N=57)	Adenomyosis with malignant transformation (N=7)	P-value
Age (years)		63 ± 9.3	65 ± 10.1	0.60
Menopause		49 (86%)	7 (100%)	0.58
Co-existing Fibroids		24 (42.1%)	1 (14.3%)	0.23
Co-existing Endometriosis		1 (1.8%)	1 (14.3%)	0.20
CA125 elevated (>35 U/ml)		8 (17.8%)	2 (33.3%)	0.58
FIGO Stage				0.57
	I	51 (89.5%)	6 (85.7%)	
	II	3 (5.3%)	0 (0%)	
	III	2 (3.5%)	1 (14.3%)	
	IV	1 (1.7%)	0 (0%)	
Tumor Grade				0.19
	1	20 (35.1%)	2 (28.6%)	
	2	33 (57.9%)	3 (42.8%)	
	3	4 (7%)	2 (28.6%)	

Concerning the survival rates, through the follow-up of these patients, there was only 1 case of recurrence, a woman who had FIGO stage III disease at the time of the diagnosis, and none death due to cancer until now.

## Discussion

Adenomyosis has been increasingly diagnosed in the last decades, due to the new non-invasive diagnostic techniques. Interestingly, there has been an ongoing search on whether its co-existence with endometrial cancer is of clinical significance, because of the nature of this benign disease. The aim of our study was to investigate whether the presence of adenomyotic tissue has an impact on the FIGO stage and therefore the prognosis of endometrial cancer. In our study, 64 (28%) out of 229 patients with endometrial cancer, had co-existing adenomyosis, which is higher compared to the reported results (18.9%) found by Mao et al. (2017). However, it was mentioned in the largest review in the literature (Habiba et al., 2018), that it is difficult to estimate the exact prevalence of the disease, due to the lack of large case-series and the different methods used by the researchers to diagnose adenomyosis. The mean age of the patients with concurrent endometrial cancer and adenomyosis was approximately 63 years old and the majority of them were postmenopausal. This finding is in accordance with the fact that endometrial cancer appears in the later life stages, but not with the fact that adenomyosis is a disease appearing earlier in reproductive age (Erkilinç et al., 2018). Also, the mean age reported in our study is higher from what the other authors found (Mao et al., 2017; Aydin et al., 2018; Erkilinç et al., 2018).

Moreover, on further analyses between the two groups, which were rather homogeneous, a statistically significant difference was found at the FIGO stage and the tumor grade. Patients with concurrent endometrial cancer and adenomyosis suffered from earlier endometrial cancer and lower grade tumors, which results in better prognosis and higher survival rates. This is in agreement with the current opinion that adenomyosis-associated endometrial cancer has a more favorable prognosis and might be a result of early diagnosis, due to the prominent symptoms of the two diseases (Habiba et al., 2018). On the other hand, the above-mentioned finding supports the idea, proposed by other authors (Koshiyama et al., 2004; Musa et al., 2012; Matsuo et al., 2014; Erkilinç et al., 2018), that adenomyosis can play a significant role as a prognostic factor in endometrial cancer, through its protective barrier effect to myometrial invasion of the tumor. Furthermore, our study included all histological subtypes, increasing the accuracy of our results, which was also suggested by Erkilinc S. et al. (2018). However, these results remain controversial, because other authors found adenomyosis as a risk factor for myometrial invasion and hence for worse prognosis ( Ismiil et al., 2007; Taneichi et al., 2014; Aydin et al., 2018). The most prominent explanation is that adenocarcinoma involving adenomyotic tissue gains an “advantage” for more aggressive spread, due to the larger contact space with the endometrium (Ismiil et al., 2007). However, it is important to mention that the study from Ismiil et al. has an important limitation, because it includes only grade I endometrial cancer.

Another important issue of our study was to further investigate if there was malignant transformation of the adenomyotic tissue, which is not fully researched, mainly due to the low incidence of this entity and the relative diagnostic problems. Out of 229 patients with endometrial cancer, 7 (3.1%) presented with malignant transformation of the adenomyotic tissue, which is accordance with the rest available literature (Kucera et al., 2011; Mao et al., 2017). Even though adenomyosis is defined as ectopic endometrial tissue inside the myometrium inside the myometrium and the pathogenetic mechanism of malignant transformation of the later is fully described (Koike et al., 2013), the etiology of malignant transformation of adenomyosis is yet not clear, but some authors propose a role of genetic and epigenetic factors (Matsuo et al., 2016). One of the main reasons for this lack of large evidence is that malignant transformation of adenomyosis has no prominent symptoms, no specific clinical, laboratory or imaging tests for its preoperative diagnosis (Mao et al., 2017). So, the diagnosis is established postoperatively, based on both Sampson’s (“Endometrial cancer (cancer of the lining of the womb) statistics | World Cancer Research Fund International,” n.d.) standard and Scott’s (Scott, 1953) supplementary criteria: no cancerous foci in endometrium or other pelvic sites, which distinguish that the malignant lesion is originated from the adenomyosis and not from the myometrial invasion / presence of benign adenomyosis around the malignant lesion / evidence of gland structure transformed for benign to malignant. The available literature on this topic is controversial, because it is based on studies with small samples. Our series is one of the biggest and showed no statistically significant differences between the two subgroups. Women suffering from adenomyosis malignant transformation did not seem to have a worse prognosis, which is in agreement with other authors (Mittal and Barwick, 1993), who argue that there is no adverse effect in the survival rates of these women. On the other hand, Matsuo et al. (2016) argues that there is a clear negative impact from the malignant transformation of the adenomyotic tissue, based on several possibilities. However, the results of the aforementioned study should be carefully interpreted, because the 46 patients with adenomyosis and malignant transformation were pooled after a review of the literature, so there is no reassurance that pathological slides were reviewed by expert pathologists, who took into consideration all the aforementioned criteria. In contrast, in our study when adenomyosis was present in the pathological reports of patients with endometrial cancer, the relevant slides were reviewed by two independent expert pathologists, who distinguish the cases with adenomyosis foci only from others with adenomyosis foci and malignant transformation, which strengthens the power of the results.

Last but not least, the possible limitations of our study are its retrospective nature and the presence of some potential confounding factors, such as comorbidities. So, further investigation is needed in order to clarify the pathologic progression of adenomyotic lesions to endometrial cancer, the associated prognosis of this disease and the estrogen status of these malignant lesions.

## Conflict of interest:

The authors declare that they have no conflict of interest. Funding: No funding is provided for the study.
